# Transboundary viral diseases of pigs, poultry and ruminants in Southeast Asia: a systematic review

**DOI:** 10.1080/01652176.2024.2397796

**Published:** 2024-08-30

**Authors:** Thi Ngan Mai, Thanh Trung Nguyen, Sinh Dang-Xuan, Hung Nguyen-Viet, Fred Unger, Hu Suk Lee

**Affiliations:** a Department of Veterinary Microbiology and Infectious Diseases, Vietnam National University of Agriculture, Hanoi, Vietnam; b Department of Pharmacology, Toxicology, Internal Medicine and Diagnostics, Vietnam National University of Agriculture, Hanoi, Vietnam; c Animal and Human Health program, International Livestock Research Institute, Hanoi, Vietnam; d College of Veterinary Medicine, Chungnam National University, Daejeon, Republic of Korea

**Keywords:** Livestock, prevalence, Southeast Asia, Systematic review, TADs

## Abstract

Livestock is a strategic part of the small-farm economy in Southeast Asia’s society, providing food income, clothing, fertilizer, and draught power. However, incidences or outbreaks of transboundary animal diseases (TADs) are due to converging factors such as the natural hazards’ aftermath, climate change, deforestation, urban growth, changing production conditions, and market chains. Therefore, this affects productivity and impacts farmers’ livelihoods with small holdings. The literature review was carried out to understand the current situation of TADs in Southeast Asia, identifying knowledge gaps to provide actions for disease control and prevention in the region. We have attempted to summarise the scientific literature in English on the prevalence data of TADs in Southeast Asia between 2011 and March 2022. Relatively few studies evaluated the distribution of TAD, where most of the studies focused on diseases that are important for international trade, such as avian influenza (AI), African swine fever (ASF), classical swine fever (CSF), foot-and-mouth disease (FMD) and Newcastle disease (ND). Traditional production systems have received little attention in such studies as they belonged to mainly smallholders. The outbreaks of ASF and lumpy skin disease (LSD) in 2019 resulted in increased research activity between 2019-2022, while the other TADs were ignored in this period. For new emerging TADs diseases such as ASF and LSD, there is only information about the first detection without prevalence information. Therefore, further epidemiological investigations are necessary to reduce the gaps in disease surveillance reporting systems and support the prevention and reduction of further outbreaks.

## 1. Introduction

Livestock production is critical to human nutrition and health in low- and middle-income countries (LMICs) (Milton 2003). Southeast Asia (SEA), the vast region of Asia situated east of the Indian subcontinent and south of China that includes 11 countries. And the Association of Southeast Asian Nations (ASEAN) an intergovernmental organization of ten Southeast Asian countries, Timor-Leste is joining ASEAN soon. In SEA, livestock represents a strategic part of the small-farm economy in the region but contributes only 15% of the agricultural gross domestic product (WOAH 2021). These animals play important roles in society, providing income and food, clothing, building materials, fertilizer and draught power (Gordon 2018). However, the presence of endemic and emerging diseases, as well as other factors (ecological and environmental changes), impact them negatively, jeopardizing their contributions.

Countries in SEA frequently experience disease emergence due to a number of converging factors: the aftermath of natural hazards, climate change, deforestation, urban growth, and changing production systems and market chains (Guha-Sapir and van Panhuis 2009). Besides commercial farms, many publications indicated that backyard and smallholder production systems are still popular in SEA (Samkol et al. 2015; Holt et al. 2019). The socioeconomic crises caused by outbreaks of avian influenza in 2004 in Asia (Webster et al. 2005) served to elevate the awareness of the wide-ranging negative impacts of infectious diseases on human health, food safety, livestock trade, and the livelihoods of poor farming communities. Globally and regionally, there have been incursions and spread of other transboundary animal diseases such as foot and mouth disease (FMD) (Edwards 2004), classical swine fever (CSF), and African swine fever (ASF) (Liu et al. 2020). The resultant losses of livestock belonging mainly to smallholders have undoubtedly indicated a major disconnect and weakness in public health and veterinary services. All these diseases may have a wildlife reservoir and may also involve domestic animals, for example, wild boar as an ASF virus reservoir.

Transboundary animal diseases (TADs) are defined as those diseases that are of significant economic, trade and food security importance for a considerable number of countries, which can quickly spread to other countries and reach epidemic proportions, and where control/management, including exclusion, requires cooperation between several countries (FAO 2004). They are more frequent in lower-middle and low-income countries. Consequently, emerging or re-emerging in these countries robs people of income and food and has a very negative impact on human health (Torres-Velez et al. 2019).

In SEA, livestock sectors, especially poultry, have grown rapidly in the past decade (Delgado et al. 2001). This increase was predominantly driven by high-income growth, rapid urbanization, and changes in dietary patterns. However, they are currently constrained by TADs such as FMD, CSF, ASF, and avian influenza, which cause high morbidity and mortality rates in livestock. Livestock in SEA is raised mainly in an extensive or backyard system (Del Rosario 2007) and is linked to several emerging diseases and has been identified as a risk factor for future emerging infections (Biswas et al. 2009). In addition, animal and human populations living in close proximity to each other are one of the important risk factors for emerging infectious diseases in SEA. Moreover, hunting practices and the increasing trend of wildlife pets in Southeast Asian countries in general also increase the risk of this threat. Animal movement and the informal livestock trade, within and between various countries in the region, are the major means of transmitting and spreading TADs (VanderWaal et al. 2020).

Rapid increases in the global human and farmed-animal population, and enormous increases in the international movement of people, animals, and livestock products expose our planet to increased risk of the outbreak of epizootic and zoonotic diseases, including potentially catastrophic pandemics such as the recent COVID-19 (Wu et al. 2022). Around 60% of all human diseases are zoonotic and so thus animal disease outbreaks also pose the potential risk of public health emergencies (Woolhouse and Gowtage-Sequeria 2005). For instance, the recent COVID-19 outbreak may be associated with wildlife harvesting, trade practices and the intensification of wildlife farming (Whitfort 2021).

TADs have the possibility to cause negative socioeconomic and public health effects. It’s necessary to understand the risk factors contributing to the spread of TADs. Further studies are also needed to improve the efficacy and cost of diagnostics and prevention measures for these diseases. Moreover, TADs of viral origin were prioritized for regional cooperation by ASEAN countries such as HPAI, FMD, CSF etc. which may have changed in the wake of the outbreak of ASF and LSD. Control of TADs has a positive impact on the livelihoods of poor farming communities, and on the regional livestock trade. This literature review aims to understand the current situation of TADs in SEA, identifying knowledge gaps to provide required actions for disease control and prevention in the region.

## 2. Materials and methods

### 2.1. Protocol and eligibility criteria

**Figure 1.**
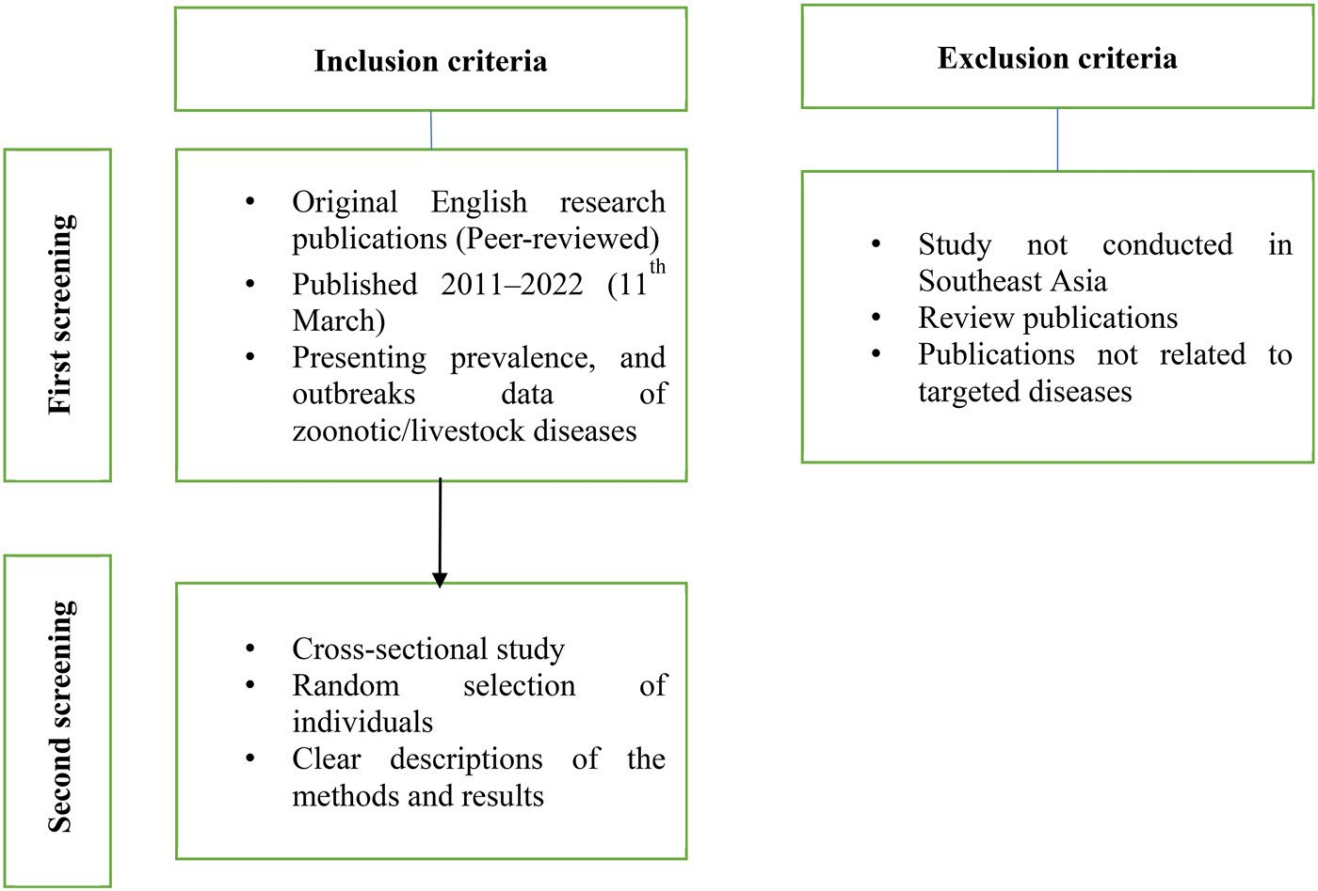
Inclusion and exclusion criteria for the first and second screening.

A protocol was developed for searching and evaluating publications, including the objective, data source, and criteria for inclusion and exclusion (Figure 1). All publications in English on the prevalence data of TADs in Southeast Asia between 2011 and March 2022 language were considered. In the first screening, the titles and abstracts were checked to see if these parts corresponded to the objective of this review. The second screening evaluated the quality of the full publication based on different inclusion and exclusion criteria (Figure 1). While the initial search resulted in many publications, a lot of them were excluded from the first screening due to the lack of information on the selection of farms and individuals. The outbreaks of ASF and lumpy skin disease (LSD) in 2019 and 2020 resulted in tremendous attention on these viruses, which could explain why other TADs in Southeast Asia were ignored in epidemiological research during this period.

All procedures were performed independently by the three authors of this manuscript and classified as ‘Yes’ or ‘No’ for inclusion. If there was a disagreement between the authors, the final collective decision was made following discussion among themselves.

### 2.2. Search strategy and syntaxes

The TADs identified by the two major international animal health entities, the Food and Agricultural Organization (FAO) and the World Organization for Animal Health (WOAH) include African horse sickness (AHS), avian influenza (AI), African swine fever (ASF), bluetongue (BT), contagious bovine pleuropneumonia (CBPP), classical swine fever (CSF), foot and mouth disease (FMD), hemorrhagic septicemia (HS), lumpy skin disease (LSD), Middle East respiratory syndrome (MERS), Newcastle disease (ND), peste des petits ruminants (PPR), rinderpest (RP), Rift Valley fever (RVF), sheeppox/goatpox (SP/GP), swine vesicular disease (SVD), and vesicular stomatitis (VS). Of these, the diseases present in Asia are ASF, BT, CBPP, CSF, FMD, HPAI, HS, LSD, ND, PPR, and SP/GP (Clemmons et al. 2021). AI is classified as low pathogenicity (LPAI) or high pathogenicity (HPAI) based on the disease caused in domestic chicken and the molecular difference between the two, which is one amino acid change in the hemagglutinin (HA) fusion cleavage site (Swayne 2009).

Publications were searched for in *PubMed*, *Web of Science*, and *Science Direct Core Collection* databases in accordance with PRISMA-established guidelines. The keywords used were divided into three parts as follows: (i) (livestock OR swine OR pig OR cattle OR buffalo OR sheep OR goat OR poultry OR duck OR chicken OR pets OR dogs OR cats OR rats); AND (ii) (Brunei OR Cambodia OR Indonesia OR Laos OR Malaysia OR Myanmar OR the Philippines OR Singapore OR Thailand OR Timor-Leste OR Vietnam OR Southeast Asia); AND (iii) (food and mouth disease OR African swine fever OR classical swine fever OR lumpy skin disease OR high pathogenic avian influenza OR transboundary animal disease). The full lists of article titles and abstracts were imported into Endnote (version X7), and duplicates were identified and removed manually.

### 2.3. Data collection process

The data extraction template included the authors, publication year, pathogen name, animal species, sample level, diagnostic method, sensitivity/specificity of the diagnostic test, study area, sample size, number of positive samples, prevalence, and 95% confidence interval (CI). In cases where several methods were applied to one sample, the highest prevalence was released. If the 95% CI of the prevalence or the number of positive animals was absent in an article, this information was derived using the data presented in the article.

### 2.4. Synthesis of results

Descriptive statistics were summarized by species like pigs, poultry, and ruminants, with the following information: pathogen, country, year of sampling, species, sample level, sample size, number positive, diagnostic test, test sensitivity/specificity, apparent prevalence (AP), 95% CI, and author (year).

## 3. Results

### 3.1. Study selection

**Figure 2.**
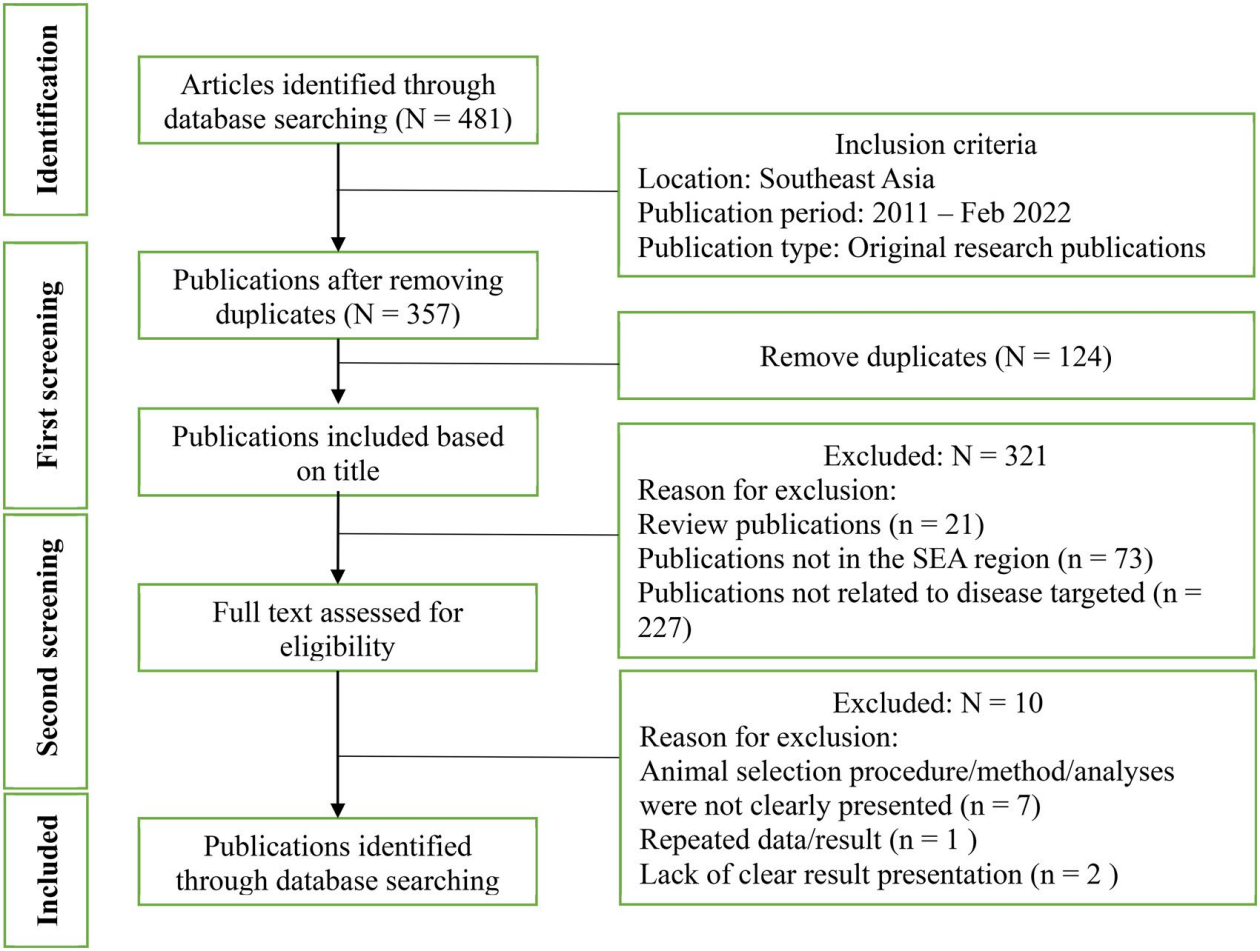
Schematic flow chart of the literature selection for the review on transboundary animal diseases in South-East Asia.

**Figure 3.**
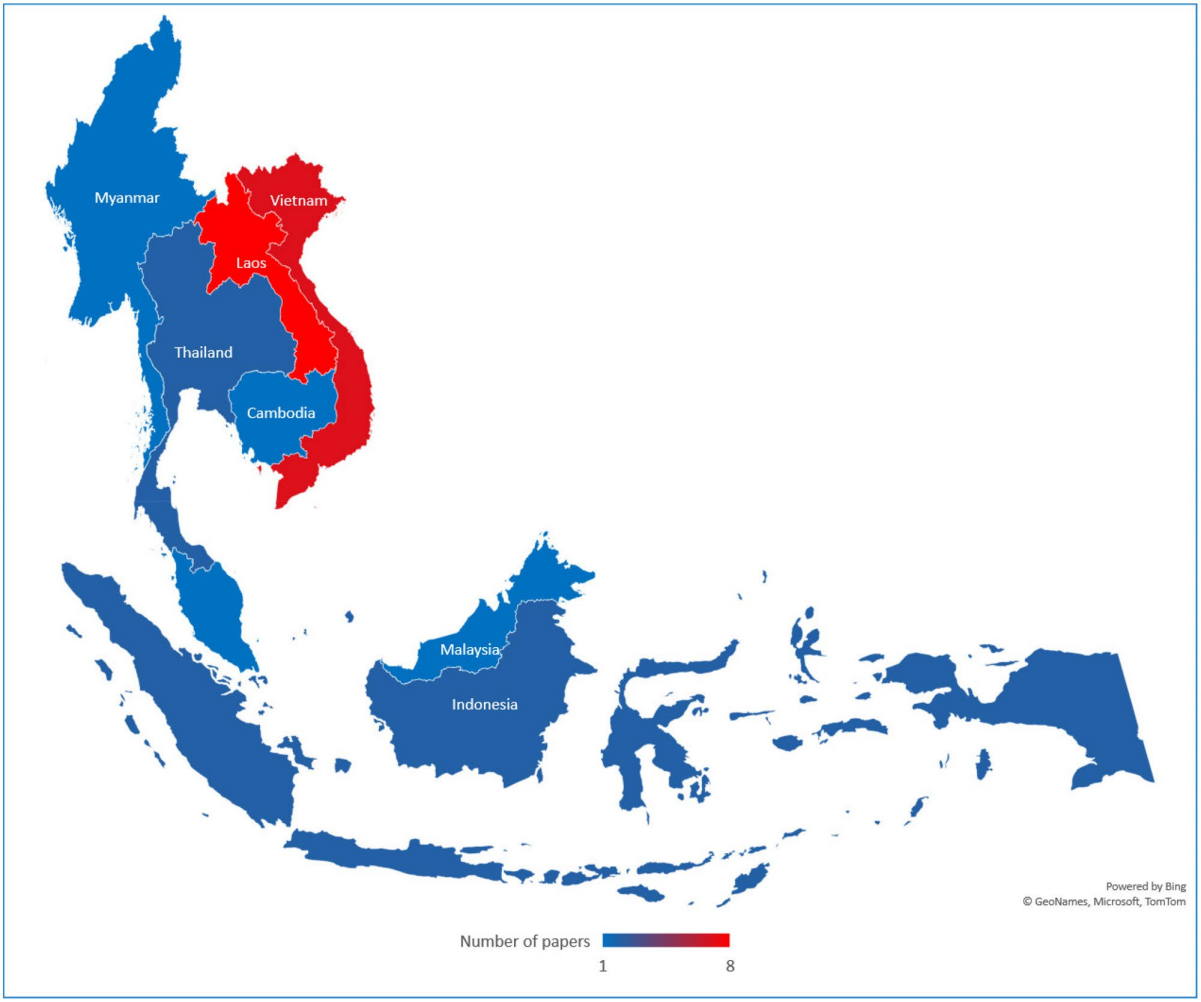
The geographical distribution of studies on transboundary animal diseases in Southeast Asia.

A total of 481 publications were retrieved from *PubMed*, *Web of Science*, and *Science Direct*. In the first screening, 124 duplicates were identified and removed, and some publications were excluded due to not being conducted in SEA (*n* = 73), review publications (*n* = 21), or not being related to the targeted diseases (*n* = 227). Thus, a total of 36 publications were included for full-text assessment in the second screening, where 10 publications were excluded because of an unclear animal selection procedure (*n* = 7), the same data being presented in two publications (*n* = 1), or the results not being presented in a clear way (*n* = 2). 26 publications were finally included (Figure 2). Nearly two-thirds of these 26 publications were based on studies conducted in Laos (*n* = 8) and Vietnam (*n* = 7). The remaining 11 publications targeted other countries (Timor-Leste, *n* = 3; Indonesia and Thailand, *n* = 2; Cambodia, Malaysia, Myanmar, and SEA, *n* = 1) (Figure 3).

### 3.2. Diseases in pigs

Eleven out of twenty-six publications that were included in the qualitative synthesis were related to pigs (Table 1).

**Table 1.** List of studies focused on transboundary animal diseases in pigs in Southeast Asia.

Pathogen	Country	Year of sampling	Species	Sample level	Sample size	Number positive	Diagnosis test	Test Se/Sp	Prevalence	95%CI	Author (Year)
ASF	Timor-Leste	2019	Pigs	Survey	436	59	LAMP	0.98/0.999	13,53	10.54–17.19	(Phillips et al. 2021)
ASF	Vietnam	2019	Pigs	First oubreak, case report	2	2	PCR	NA	NA	NA	(Nga et al. 2020)
ASF	Laos	2019	Pigs	Evidence of present	107	57	Pen-side rapid test	0.65/0.76	NA	NA	(Matsumoto et al. 2020)
ASF	Indonesia	2019–2022	Pigs	Outbreak investigation	29	16	Real-time PCR	NA	NA	NA	(Dharmayanti et al. 2021)
ASF	Malaysia	2021	Pigs	First detection	14	14	Real-time PCR	NA	NA	NA	(Khoo et al. 2021)
ASF	Laos	2019, 2020	wild boar	Passive reporting	5	4	Real-time PCR	NA	NA	NA	(Denstedt et al. 2021)
ASF	Vietnam	2019, 2020	wild boar	Passive reporting	3	2	Real-time PCR	NA	NA	NA	(Denstedt et al. 2021)
ASF	Cambodia	2019, 2020	wild boar	Passive reporting	10	0	Real-time PCR	NA	NA	NA	(Denstedt et al. 2021)
CSF	Indonesia	2010	Pigs	Cross-sectional study	2160	341	ELISA	0.98/0.99; 0.91/1	15.8	14.3–17.4	(Sawford et al. 2015)
CSF	Laos	2011	Pigs	Cross-sectional study	631	80	ELISA	NA	12.6	10.23–15.59	(Holt et al. 2019)
CSF	Timor-Leste	2013	Pigs	Cross-sectional study	720	225	ELISA	0.91/1	31.3	27.9–34.8	(Sawford et al. 2015)
CSF	Thailand	2016–2017	Pigs	Cross-sectional study	237	21	ELISA	NA	9.0	6.0–13.0	(Chumsang et al. 2021)
FMD	Laos	2011	Pigs	Cross-sectional study	609	119	ELISA	NA	19.5	16.5–23.0	(Holt et al. 2019)
FMD	Laos	2019–2020	Pigs	Surveillance	597	8	ELISA	0.917/0.995	1.3	0.7–2.6	(Siengsanan-Lamont et al. 2021)

Out of these, six publications investigated ASF (*n* = 6) but only one study gave information on prevalence; the rest just provided information about the first detection. The outbreaks of ASF in SEA since 2019 resulted in enormous attention on ASF, therefore many studies have focused on ASF since 2019. Four publications showed evidence of the first detection in Vietnam (Nga et al. 2020), Laos (Matsumoto et al. 2020), Indonesia (Dharmayanti et al. 2021), and Malaysia (Khoo et al. 2021). The authors concluded that ASFV in Malaysia was similar to ASFV in Indonesia, Vietnam and China (Khoo et al. 2021). In Vietnam, the ASFV strain isolated from the first outbreaks was 100% similar to the genotype II (p72) isolates from Georgia in 2007 and China in 2018 (Nga et al. 2020). Phylogenetic and sequences analysis of the ASFV in the samples from both North Sumatra and West Java in Indonesia were identical and also identical to another genotype II ASFV from domestic pigs in Vietnam, China and Russia, indicating a common source of infection belonging to the p72 genotype II and serogroup 8 (Dharmayanti et al. 2021). There was evidence of ASF in Laos from mid-2019 (Matsumoto et al. 2020). The author used ASF samples submitted to the Laos National Animal Health Laboratory between June and December 2019 to evaluate the pen-side rapid diagnostic tests. This study gives proof of the use of low-cost rapid ASF diagnostic tests in the field where resources may be limited (Matsumoto et al. 2020).

Even though there was neither prevalence nor first detection information on ASF in other Southeast Asian countries such as the Philippines and Cambodia, there was evidence of ASF in these countries (Lokhandwala et al. 2019; WOAH 2019). One article indicated the detection of ASFV in free-ranging wild boars in SEA (Denstedt et al. 2021). The authors indicated an extensive overlap between wild boar habitats and domestic pig areas around villages bordering forests in Vietnam, Laos and Cambodia, creating a high-risk interface for viral spillover between domestic pig and wild boar populations (Denstedt et al. 2021). This study is the first report of ASF in wild boars in SEA, a probable spillover from domestic pigs. In addition, it is still unknown whether wild boars can maintain the virus in a wild boar-habitat cycle in SEA as is the case in Europe, or whether they play an epidemiological role in transmitting the virus back to domestic pigs. Therefore, the authors highlighted the importance of early reporting and monitoring of ASF in wild boars to enable the implementation of appropriate biosecurity measures, and identifying the ASF transmission pathways from domestic pigs within the Southeast Asian context to reduce the impact of ASF.

Out of six ASF publications, only one targeted Timor-Leste with an AP of 13.53% at the animal level and village-level prevalence of 16/48 = 34% (95% CI 22–48%) (Phillips et al. 2021). In this study, the LAMP assay was proven to be a robust, highly specific and sensitive laboratory test for ASF suitable for use in the field and where there are limited laboratory facilities. Timor-Leste confirmed an outbreak of ASF in September 2019 (Phillips et al. 2021). Pigs are the second most common type of livestock kept by villagers in Timor-Leste and represent a traditionally important source of income for householders. The results of the prevalence survey allowed delineation of the extent of the ASF incursion and the introduction of a disease response strategy to limit the spread of ASF and assist in the recovery of the pig population in Timor-Leste.

Of the remaining publications, four targeted CSF (*n* = 4, AP 9,0–31,3%) (Sawford et al. 2015; 2015; Holt et al. 2019; Chumsang et al. 2021), and two focused on FMD (*n* = 2, AP = 1,3–19,5) (Holt et al. 2019; Siengsanan-Lamont et al. 2021), with one of these publications covering both FMD and CSF (Holt et al. 2019). The prevalence of CSFV antibodies among backyard pigs in Chiang Mai, Thailand, in 2016–2017 was indicated at 14% (95% CI: 9–20) at the swine level by a cross-sectional serological study (Chumsang et al. 2021). In the other two studies, CSFV seroprevalence was estimated at 17.5% and 34.4% from cross-sectional surveys in four islands of Indonesia and three districts of Timor-Leste (bordering Indonesia), respectively (Sawford, do Karmo, et al. 2015; Sawford, Geong, et al. 2015).

A total of 1,280 sera in six provinces of Laos were collected and tested for FMD non-structural protein (NSP) antibodies to indicate natural infections from March to December 2019 (Siengsanan-Lamont et al. 2021). This study sampled cattle, buffaloes, and pigs and found the seropositivity rate for FMD NSP antibodies to be 44.6%, 35.0% and 1.3%, respectively (Siengsanan-Lamont et al. 2021). Serotype-specific antibody ELISA for 44 NSP antibody-positive samples revealed evidence of FMD serotypes O and A virus circulation in Laos. The passive abattoir survey provided information on the FMD virus’s previous exposure, the geographic locations of the animals, and FMD virus circulation, which is crucial to an effective control program.

The same study investigated antibodies of CSF and FMD in pigs in representative upland and lowland provinces in Laos and found an AP of CSF (11.2%), FMD O (17.2%), and FMD Asia 1 (3.5%) (Holt et al. 2019). Vaccination coverage for FMD and CSF was low and there was a lack of national funding for livestock disease control in 2011.

### 3.3. Diseases in poultry

Seven publications on poultry were included in the qualitative synthesis, all of them targeting chickens and ducks (Table 2).

**Table 2.** List of studies focused on transboundary animal diseases in poultry in Southeast Asia.

Pathogen	Country	Year of sampling	Species	Sample level	Sample size	Number positive	Diagnosis test	Test Se/Sp	Prevalence	95%CI	Author (Year)
ND	Timor-Leste	12/2008–8/2009	Chicken	Animal	1674	211	Haemaggluti- nation inhibition (HI) test	NA	12.6	10.5–14.7	(Serrão et al. 2012)
ND	Timor-Leste	12/2008–8/2009	Chicken	Flocks	300	106	Haemaggluti- nation inhibition (HI) test	NA	35.3	29.9–40.7	(Serrão et al. 2012)
ND	Laos	2015	Chicken	Seroprevalence	123	107	ELISA	NA	86.9	79.4–92.2	(Pauly et al. 2019)
ND	Vietnam	2017–2019	Chicken	Animal	75	49	ELISA	NA	65.3	53.4–75.7	(Van et al. 2020)
AI (HPAI)	Vietnam	2005–2006	Chicken	Case study	1601	52	Seroneutralization test	NA	3.25	2.39–4.11	(Trevennec et al. 2011)
AI (HPAI)	Vietnam	2012	Ducks	Duck level	12480	82	Real-time RT-PCR	1.0/1.0	0.67	0.53–0.83	(Phan et al. 2013)
AI (HPAI)	Vietnam	2016–2017	Ducks	Animal	156	0	Real-time RT-PCR	NA	0	0–3.0	(Thanh et al. 2017)
AI (HPAI)	Vietnam	2016–2018	Chicken	Animal	96	0	Real-time RT-PCR	NA	0	0–4.8	(Thanh et al. 2017)
AI (HPAI)	Vietnam	2017–2019	Chicken	Flocks	61	3	RT-PCR	NA	4.9	1.3–14.6	(Van et al. 2020)
AI	Vietnam	2005–2006	Chicken	Case study	1601	155	ELISA	0.987/0.9872	9.68	8.3–11.3	(Trevennec et al. 2011)
AI	Timor-Leste	2009	Chicken	Animal	1134	4	ELISA	NA	0.4	0.0–0.7	(Serrão et al. 2012)
AI	Vietnam	2017	Chicken	Animal	200	55	Real-time RT-PCR	NA	27.5	21.6–34.3	(Tran et al. 2020)
AI	Vietnam	2017	Ducks	Animal	202	50	Real-time RT-PCR	NA	24.48	19.1–31.4	(Tran et al. 2020)

Three of the publications investigated Newcastle disease (ND) (*n* = 3, AP of 12.6–86.9% at the animal level and 4.9–35.3 at the flock level) (Serrão et al. 2012; Pauly et al. 2019; Van et al. 2020). The publications on NDV were from 2008 to 2019. In Laos, despite serological evidence of NDV circulation (86.9%) with no difference in seroprevalence between juvenile and adult birds, virus RNA was detected in none of the 123 swab samples (Pauly et al. 2019).

Another disease of global importance among poultry is AI. Six publications investigated AI and HPAI with AP of 0.4–27.5% and 0–3.25% at the animal level and 4.9% at the flock level for HPAI, respectively (Trevennec et al. 2011; Serrão et al. 2012; Phan et al. 2013; Thanh et al. 2017; Tran et al. 2020; Van et al. 2020). Most of the AI studies were conducted in Vietnam. One study targeted 2005 ethnic minority households in Northern Ha Giang Province, located on the Chinese border, where the seroprevalence of AI virus was estimated at 7.2% (Trevennec et al. 2011). The H5 and H9 subtypes had a seroprevalence of 3.25% and 1.12%, respectively (Trevennec et al. 2011). Another study was conducted at live bird markets/slaughter points in five provinces in the Red River, Mekong Delta, and central Vietnam in January and May 2011. It indicated the highest prevalence (6.6%) of AI in the Mekong Delta and no H5N1 detection in the two Red River provinces (Phan et al. 2013). The prevalence of AI virus among chicken and duck samples was 27.5% and 24.8%, respectively (Tran et al. 2020). In this study, 402 positive chicken samples with A/H5 contained 99% nucleotide similarity with the H5N6 reference strain, suggesting that while the presence of LPAI virus was predominant, potential risks of the appearance of HPAI virus in the east-west boundary in Vietnam should be a concern and studied further (Tran et al. 2020). The presence of various strains of LPAI, a new strain of H5N6 HPAI, and the potential risks of these strain recombinants in the bordering area suggested that HPAI is being spread silently across geographical areas (Tran et al. 2020). Another study indicated that the circulation of subtype H5 influenza viruses on smallholder poultry farms was low (0/378 samples) in Ca Mau, Vietnam in 2016 (Thanh et al. 2017).

One longitudinal study to monitor the prevalence of antibodies against both ND virus and AI virus in chickens was conducted in four districts of Timor-Leste during three sampling periods from December 2008 to August 2009 (Serrão et al. 2012). None of the birds enrolled in the study was vaccinated against ND or AI. The bird-level ND seroprevalence was 12.6%. Only four samples tested AI positive in a total of 1,134 samples, indicating a bird-level seroprevalence level for AI of 0.4% (CI 0.0–0.7%). These AI-positive samples were further tested for subtypes H5N1, H5N3, H7N3, and H9N2, but all tested negative, suggesting that the influenza antibodies in those four birds resulted from exposure to LPAI viruses of different H subtypes (Serrão et al. 2012). The other study targeted both ND and AI in Vietnamese small-scale chicken flocks and confirmed severe HPAI H5N1 infection in three flocks, one of which had previously been vaccinated with an injectable inactivated H5N1-based vaccine (Van et al. 2020). Overall, the majority of the studies on poultry (*n* = 7) were conducted on ND and AI in Vietnam and Timor-Leste.

### 3.4. Diseases in ruminants

A total of eight publications on ruminants were included, most of them related to FMD (Table 3).

**Table 3.** List of studies focused on transboundary animal diseases in ruminants in Southeast Asia.

Pathogen	Country	Year of sampling	Species	Sample level	Sample size	Number positive	Diagnosis test	Test Se/Sp	Prevalence	95%CI	Author (Year)
FMD	Cambodia	2010	Cattle, buffalo	Village-level seroprevalence	38	25	ELISA	0.926/0.961	65.8	48.6–79.9	(Bellet et al. 2012)
FMD	Myanmar	2012–2016	Cattle	village-level seroprevalence	160	126	ELISA	1/0.95-0.99	79	72–84	(van Andel et al. 2020)
FMD	Laos	2017–2018	Goats	Animals	591	77	ELISA	NA	13.0	10.3–15.7	(Singanallur et al. 2020)
FMD	Laos	2019	Cattle	Cross-sectional study	498	219	ELISA	1/0.998	44.0	39.6–48.5	(MacPhillamy et al. 2022)
FMD	Laos	2019	Goats	Cross-sectional study	19	4	ELISA	1/0.998	21.1	7.0–46.1	(MacPhillamy et al. 2022)
FMD	Laos	2019	Buffalo	Cross-sectional study	104	45	ELISA	1/0.998	43.3	33.7–53.3	(MacPhillamy et al. 2022)
FMD	Laos	2019–2020	Buffalo	Animal. Surveillance	214	75	ELISA	0.917/0.995	35.0	29.0–41.7	(Siengsanan-Lamont et al. 2021)
FMD	Laos	2019–2020	Cattle	Animal. Surveillance	469	209	ELISA	0.917/0.995	44.6	40.1–49.1	(Siengsanan-Lamont et al. 2021)
LSD	Vietnam	2020	Cattle	Oubreak, first information	1	1	real-time PCR	NA	NA	NA	(Tran et al. 2021)
LSD	Thailand	2021	Cattle	Oubreak, first information	1888	10	Clinical signs, real-time PCR, isolation	NA	NA	NA	(Arjkumpa et al. 2021)
PPR	Laos	2016–2017	Goats	Animals	1072	23	ELISA	NA	2.2	1.4–3.2	(Burns et al. 2019)

Five of the publications investigated FMD with an AP of 13.0–44.6% at the animal level and a village-level prevalence of 65.8–79% (Bellet et al. 2012; Singanallur et al. 2020; van Andel et al. 2020; Siengsanan-Lamont et al. 2021; MacPhillamy et al. 2022). One of these studies also sampled buffaloes, cattle, and pigs (Siengsanan-Lamont et al. 2021). Two a publications showed evidence of the first detection of LSD in Vietnam and Thailand (Arjkumpa et al. 2021; Tran et al. 2021). Only one study investigated PPR in Laos, with an AP of 2.2% (95% CI: 1.4–3.2) (Burns et al. 2019). Overall, the majority of the studies on ruminants (*n* = 8) were conducted on FMD and LSD and most of the FMD studies were targeted at Laos. Results from another two studies in Cambodia and Myanmar that evaluated the village-level seroprevalence confirmed the strong enzootic transmission of FMD in these countries because of the high prevalence (Bellet et al. 2012; van Andel et al. 2020).

For LSD, two studies provided information about the first detection of LSD in Vietnam and Thailand. LSDV isolated in the first outbreak in Vietnam was 100% identical to viruses isolated in China (2019) based on the p32 and RP030 genes and very close to the virus isolated in Russia (2017) based on the p32, RP030, thymidine kinase, and ORF103 genes (Tran et al. 2021). In Thailand, an LSD outbreak involving beef cattle farms in the Northeastern Region on 29 March 2021 was suspected to be the first occurrence of LSD (Arjkumpa et al. 2021). Phylogenetic analysis of the LSDV fusion protein gene showed that the sequence of the Thai isolate was similar to the currently circulating isolates from Russia/2019, India/2019 and Kenya/2019, sharing 99.8%–100% nucleotide identity (Arjkumpa et al. 2021). The Thai Department of Livestock Development has implemented considerable control measures, including strict quarantine and movement control measures, zoning, surveillance outside/within the protection zone, vector control, and disinfection in the outbreak area (Arjkumpa et al. 2021).

Only one study showed serological evidence of PPR in SEA, an economically important transboundary viral disease of sheep and goats (Burns et al. 2019). A total of 1,072 serum samples were collected across five provinces in Laos and tested for antibody response to PPRV ELISA, resulting in an AP of 2.2% (Burns et al. 2019).

### 3.5. Transmission risk factors and prevention measures

#### Transmission risk factors

Out of 11 publications related to pigs, there were five publications that included information about transmission risk factors of CSF and FMD in Indonesia, Laos, Timor-Leste and Thailand (Table 4). Older pigs, penned housing in the dry season, CSFV vaccination status, the sudden death of pigs in the last 12 months, sickness in the last three months, and using artificial insemination for breeding were associated with CSFV status (Sawford et al. 2015; 2015; Holt et al. 2019; Chumsang et al. 2021). Age, using *sanaam* (a communal area where pigs are kept for some time of the year), penned housing in the dry season and geographical location (high density and transit routes for transboundary animal movement) were related to FMD (Holt et al. 2019; Siengsanan-Lamont et al. 2021).

**Table 4.** Summary of transmission risk factors on transboundary animal diseases in pigs, poultry and ruminants in Southeast Asia.

Pathogen	Country	Species	Risk factors	OR/RR	95%CI	Author (Year)
CSF	Indonesia	Pigs	Age (≥ 12 months)	2.52	1.40–4.54	(Sawford et al. 2015)
			Vaccinated for CSFV	3.17	1.68–5.98	
CSF	Laos	Pigs	Housing in dry season (penned)	0.28	0.08–0.80	(Holt et al. 2019)
CSF	Timor-Leste	Pigs	Age (≥ 24 months)	3.76	1.65–8.54	(Sawford et al. 2015)
			Vaccinated for CSFV	2.53	1.28–5.02	
			Sudden death of pigs in the last 12 months	2.39	1.15–4.99	
			Sick in the last three months	0.18	0.04–0.96	
CSF	Thailand	Pigs	Using artificial insemination for breeding	7.7	1.49–39.9	(Chumsang et al. 2021)
FMD	Laos	Pigs	Housing in dry season (penned)	0.27	0.11–0.67	(Holt et al. 2019)
			Using *sanaam*	3.34	1.23–9.32	
FMD	Laos	Pigs, cattle, buffalo	Less than 1 year old	2.5	1.4–4.4	(Siengsanan-Lamont et al. 2021)
			Khammouane Province to Champasak Province	4.5	1.1–18.7	
			Xiengkhouang Province to Champasak Province	2.4	1.4–4.1	
HPAI	Vietnam	Chickens	Age of chickens	NA	NA	(Van et al. 2020)
AI	Vietnam	Chickens	Number of inhabitants in the village	+24.9%	NA	(Trevennec et al. 2011)
			Regression residuals of the distance to the national road	−1%	NA	
HPAI	Vietnam	Ducks	Trader sells directly to the public	1.88	1.03–3.45	(Phan et al. 2013)
			Ducks sold by trader originate from >1 farms	2.43	1.33–4.47	
			Ducks aged >6–12 months	5.01	1.95–12.8	
FMD	Laos	Goats	Age 13–24 months	9.97	3.32–29.89	(Singanallur et al. 2020)
			Age >24 months	12.68	3.99–40.30	
			Sex (female)	0.29	0.10–0.83	
FMD	Laos	Cattle, buffalo	Animal has had suspected case of FMD	1.96	1.06–3.65	(MacPhillamy et al. 2022)

OR: odd ratio; RR: relative risk; CI: confident interval.

For poultry, three of seven publications had information about transmission risk factors of AI and HPAI in chickens and ducks in Vietnam (Trevennec et al. 2011; Phan et al. 2013; Van et al. 2020). Age, number of inhabitants in the village, distance to the national road, the trader selling directly to the public, and ducks bought by a trader on the same day coming from more than one farm were associated with AI and HPAI in Vietnam (Table 4). Three of the eight a publications included information on transmission risk factors of FMD in ruminants in Laos, which were age, sex, geographical location (high density and transit routes for transboundary animal movement), and animal having had a suspected case of FMD (Singanallur et al. 2020; Siengsanan-Lamont et al. 2021; MacPhillamy et al. 2022).

#### Prevention measures

From the ASF result survey, Timor-Leste has implemented a staged disease response that includes some movement controls, improving biosecurity awareness and practices amongst village pig owners, further sampling to ascertain the effectiveness of the implemented disease control measures, and further research and training to enable the most effective use of available laboratory resources and in-country animal health staff (Phillips et al. 2021). Passive reporting has proven an effective method of detecting ASF in wild boars. Systematic long-term surveillance to monitor ASF in wild boars, including both passive reporting and targeted study designs, is needed to further investigate the prevalence and potential circulation of ASFV in wild boars throughout Cambodia, Laos, Vietnam and the rest of SEA (Denstedt et al. 2021). Further investigation into sustainable low-cost control strategies for CSF and FMD in Laos is encouraged, with farmers engaging and promoting good biosecurity practices by increasing awareness of disease transmission and prevention (Holt et al. 2019). In addition, the results of CSFV circulated among backyard pigs in Chiang Mai, Thailand, suggest that effective control measures need to be prepared and implemented, including the strict regulation of pig imports as a source of the viruses, along with effective animal quarantine, policies and appropriate vaccination programs (Chumsang et al. 2021). Using syndromic surveillance with sample collection in targeted areas, choice of diagnostic tests, and possible use of surveillance involving data submission by mobile phones were recommended for an effective monitoring system for not only FMD but also for other zoonotic, transboundary diseases (Siengsanan-Lamont et al. 2021).

In poultry, to reduce the risk and improve effective control of HPAI as well as AIV infection, improved biosecurity, bird containment, separation of poultry species and sick birds, tailored vaccination programs, continuous surveillance, and improving the quality of day-old chicks are warranted (Pauly et al. 2019; Van et al. 2020). In addition, the survey of larger farm-level studies over time should be implemented as a tool to evaluate progress in HPAI control (Phan et al. 2013; Thanh et al. 2017). Due to the potential risk of the appearance of the HPAI virus in the east-west boundary in Vietnam, further studies should be conducted and prevention activities implemented in Vietnam as well as other Asian countries (Tran et al. 2020). The first ND and AI survey in Timor-Leste has provided valuable information that will strengthen veterinary services and surveillance work in this young country (Serrão et al. 2012).

For ruminants, the results on FMD prevalence in Myanmar suggest that verbal reports should be integrated into active FMD surveillance programs in developing countries (van Andel et al. 2020). Continued sero-surveillance for FMD in goats is recommended to improve our understanding of their role in the epidemiology of FMD in the region and to extend support to FMD control decisions, particularly regarding vaccination (Singanallur et al. 2020). Laos requires ongoing support from donor agencies to improve the current animal disease surveillance system and implement effective FMD control strategies (MacPhillamy et al. 2022). The results of PPR surveillance in goats in Laos suggest that further surveillance is needed for this country to participate in the global eradication of PPRV and to ensure that the Laos veterinary sector can respond to a PPR crisis (Burns et al. 2019).

## 4. Discussion

TADs are highly contagious epidemic diseases that can spread extremely rapidly, irrespective of national borders. They cause high rates of death and disease in animals, resulting in serious socio-economic and sometimes public health consequences while constituting a constant threat to the livelihoods of livestock farmers.

In pigs, seroprevalence estimates for CSFV varied widely across areas. The findings indicate that infection with CSFV remains a significant concern. Effective control measures need to be prepared and implemented, and these should include the strict regulation of pig imports along with effective animal quarantine and appropriate vaccination policies (Chumsang et al. 2021). Moreover, Manggarai Barat, a district of Flores Island, had antibody CSFV-positive pigs but no clinical cases had been reported in this area (Sawford et al. 2015). Therefore, further research involving antigen detection and in-depth investigation of suspected cases over a period of time is needed (Sawford et al. 2015; 2015). Due to limited study areas and time periods in FMD studies, it is difficult to determine whether these results are representative of SEA as a whole.

Besides CSF and FMD, ASF has been present in many SEA countries such as Vietnam, Indonesia, Malaysia, Laos, Cambodia and Timor-Leste since 2019 (Matsumoto et al. 2020; Nga et al. 2020; Dharmayanti et al. 2021; Khoo et al. 2021; Phillips et al. 2021). Even though there is no publication on ASF in the the Philippines and Cambodia, there is evidence of ASF in these countries (Lokhandwala et al. 2019; WOAH 2019). No papers have been published to evaluate the prevalence of ASF despite the survey program in 2019 in Timor-Leste (Phillips et al. 2021). After the incursion in mainland China in 2018, ASFV has spread rapidly throughout numerous Southeast Asian countries since early 2019. Systematic long-term surveillance to ­monitor ASF is needed to further investigate the prevalence and potential circulation of ASFV in pigs throughout SEA to reduce the gaps in disease surveillance and reporting systems as well as to support the prevention and reduction of further outbreaks. The completion of a prevalence survey to inform any jurisdictional response agency on disease distribution is a crucial step in planning any response to a new disease incursion. Swine disease control in SEA needs to be enhanced if pig production is to survive in the long term, especially with regard to ASF (Kedkovid et al. 2020).

Most of the studies on poultry diseases in the current review targeted AI or ND, especially in Vietnam, and the most recent publications for both pathogens were in the period 2017 to 2019. One of the studies indicated that chickens in Timor-Leste were exposed to ND virus but no evidence of infection with HPAI viruses was detected (Serrão et al. 2012). Another study in Laos suggested that applying an ELISA with a broader host range would be important to assess the role of ducks in NDV epidemiology (Pauly et al. 2019). The study targeting both ND and AI confirmed that besides vaccination, it was necessary to step up on-farm biosecurity practices because these vaccines are widely available (Van et al. 2020).

Subtype H5N1 avian influenza viruses, with both high pathogenicity and low pathogenicity, have been enzootic in Vietnam since 2001. One AI study indicated that the number of inhabitants in a village and the distance to the main national road were the most influential risk factors for AIV infection in Vietnam, and high-risk clusters were located along the road leading to China. The author suggested that AIV spread through commercial poultry exchanges and a possible introduction of AIV from southern China (Trevennec et al. 2011). The results of other AI studies in Vietnam recommended that market surveys be implemented over time as a tool to evaluate progress in HPAI control (Phan et al. 2013). Due to the potential risks of the appearance of the HPAI virus in the east-west boundary in Vietnam, further studies should be conducted and prevention activities implemented in Vietnam as well as in other Asian countries (Tran et al. 2020). These findings suggested that HPAI is being spread silently across geographical areas. This issue may be partly attributable to the lack of encouragement for reporting and containment efforts. Additionally, there is either no compensation or limited compensation available, and implementing biosecurity measures may not be feasible for smallholders. Previous studies in Laos and China reported that the new H5N6 HPAI virus was a reassortment of H5N1 and H6N6 viruses (Wong et al. 2015; Jiao et al. 2016). Other studies indicated that the circulation of subtype H5 influenza viruses was low in the Mekong Delta region in 2016, suggesting that larger farm-level studies should be planned (Thanh et al. 2017). All the publications focused on two diseases of global importance, AI and ND, indicating that these diseases continue to be a threat to the livestock industry in SEA.

For ruminants, most of the research has been conducted on FMD. Three publications targeted FMD in ruminants in Laos from 2017 to 2020 (Singanallur et al. 2020; Siengsanan-Lamont et al. 2021; MacPhillamy et al. 2022). The prevalence reported in goats in the 2017–2018 period was nearly two times lower than the prevalence reported in the study in 2019 (Singanallur et al. 2020; MacPhillamy et al. 2022). The prevalence reported in cattle was similar to the prevalence reported in buffalo in 2019 (MacPhillamy et al. 2022). Contrariwise, another study conducted between 2019 and 2020 showed that the prevalence reported in cattle was higher than the prevalence reported in buffaloes (Siengsanan-Lamont et al. 2021). Data collected between 2012 and 2016 in southern Laos revealed an FMD seroprevalence of more than 50% in adult ruminants while an active survey in Xiengkhouang Province in 2017 demonstrated 33.2% seroprevalence (Siengsanan-Lamont et al. 2021). The results from these studies showed that the change in prevalence between 2017 and 2020 needs to be followed up by further epidemiological studies in order to take appropriate action to prevent further infection. Pigs are the amplifying hosts, cattle are indicator hosts, and small ruminants are maintenance hosts of FMDV which may have an impact on the epidemiology of FMD.

In Southeast Asian countries, FMD outbreaks may have been controlled and prevented due to the execution of vaccination campaigns under the South-East Asia Foot and Mouth Disease (SEAFMD) program, now known as the South-East Asia and China Foot and Mouth Disease (SEACFMD) campaign. On the other hand, even though FMD is ranked second on the list of priority diseases, it seems that livestock owners see no benefit in reporting it since the disease causes low direct losses. Therefore, studies on FMD are of little interest.

No publications were found in the current review with prevalence information on LSD. After the first detection in Thailand, another study used clinical symptoms to identify 293 LSD outbreak farms in four different districts between March and the first week of April 2021 in the same country (Arjkumpa et al. 2021). Enhancing and strengthening strategies to control the potential further spread of LSD is urgent. Follow-up epidemiological studies are necessary to gain a better understanding of the disease’s characteristics and transmission dynamics.

Of the 26 publications included in the analysis, only one study targeted PPR highlights. The sampled Laos goat population is highly likely to be naïve to PPRV and therefore at risk of an outbreak, possibly due to the transboundary incursion of livestock from PPR-endemic China. However, further study is required to provide evidence of a PPR-free status in small ruminants, especially in support of global PPR eradication programs (Burns et al. 2019). Because there was only one study investigating PPR, there is a clear need for further epidemiological research to determine how widespread the disease is in the ruminant population and to understand the associated risk factors.

There was no information about the presence of other TADs such as BT, CBPP, HS and SP/GP in SEA during the search period of this review. Therefore, a further epidemiological investigation is necessary to reduce the gaps in disease surveillance and reporting systems as well as to support the prevention and reduction of further outbreaks.

Of the 26 publications included in the analysis, six focused on AI, an important TAD that can infect humans. Most of these AI publications were conducted in Vietnam indicating that AI is still a major concern for poultry livestock in Vietnam.

Another reason for discrepancies in the numbers of published papers in each country could be that some TADs seem to be under surveillance and vaccination under control or livestock owners do not see any benefit in reporting certain diseases. Therefore, a further epidemiological study is necessary to reduce the gaps in disease surveillance and reporting systems as well as to support the prevention and reduction of further outbreaks.

The popularity of backyard and smallholder production systems in SEA contributes to the high risk of TAD outbreaks in livestock due to the lower biosecurity level (Thanh et al. 2017; Nga et al. 2020; Van et al. 2020; Chumsang et al. 2021; Phillips et al. 2021) as well as challenges in movement control. Moreover, anthropogenic elements play an important role in the transmission of TADs which are often omitted. For example, there are approximately three million households in Vietnam involved in pig production, of which more than 70% are smallholders. The first case of ASF was diagnosed on a small farm and during the first epidemic year, most of the cases were on small farms (Mai et al. 2022). Therefore, it is necessary to enhance knowledge and awareness of biosecurity practices for the control of TADs among smallholder farmers.

The current review has the limitation of only including publications published in English. However, the relatively low number highlights the need for more research to be carried out on TADs in SEA.

## 5. Conclusion

Relatively few studies were found to evaluate the distribution of TADS in SEA; most focused on important diseases for international trade such as FMD, CSF, ASF, AI, and ND. Also, the traditional production systems have received little attention due to livestock belonging mainly to smallholders. This affects productivity and impacts the livelihoods of these farmers. In addition, the socio-economic impacts of TADs can be complex and extend beyond the immediate effects on directly affected producers. These impacts include effects on prices and markets, trade, food security and nutrition, health and the environment, and financial costs. It is necessary for stakeholders to collaborate at regional and sub-regional levels, combining their specific strengths to achieve the capacity required to address priority TADs using the One Health approach.

For new emerging TADs such as ASF and LSD, there is only information about the first detection. Therefore, further epidemiological investigation is necessary to reduce the gaps in disease surveillance and reporting systems as well as to support the prevention and reduction of further outbreaks. Therefore, a high level of political commitment and innovative approaches are necessary, and the ASEAN platform can be utilized for this purpose. For example, regional cooperation in the surveillance, prevention, and control of viral Transboundary Animal Diseases (TADs), particularly those designated as priorities, is essential. Establishing a vaccine bank for TADs of regional importance, such as HPAI, FMD, PPR, and LSD, would be a strategic approach to negotiating better prices, ensuring quality control, and synchronizing vaccination strategies.

## Data Availability

The data that support the findings of this study are available from the corresponding author upon reasonable request.
